# Increased sensitivity to apoptosis upon endoplasmic reticulum stress-induced activation of the unfolded protein response in chemotherapy-resistant malignant pleural mesothelioma

**DOI:** 10.1038/s41416-018-0145-3

**Published:** 2018-06-20

**Authors:** Duo Xu, Shun-Qing Liang, Haitang Yang, Ursina Lüthi, Carsten Riether, Sabina Berezowska, Thomas M. Marti, Sean R. R. Hall, Rémy Bruggmann, Gregor J. Kocher, Ralph A. Schmid, Ren-Wang Peng

**Affiliations:** 1 Division of General Thoracic Surgery, Inselspital, Bern University Hospital, Bern, Switzerland; 2 Department of Medical Oncology, Inselspital, Bern University Hospital, Bern, Switzerland; 3 Department for BioMedical Research, University of Bern, Bern, Switzerland; 4 Institute of Pathology, University of Bern, Bern, Switzerland; 5 Interfaculty Bioinformatics Unit, University of Bern, Bern, Switzerland

**Keywords:** Mesothelioma, Translational research

## Abstract

**Background:**

Standard treatment for advanced malignant pleural mesothelioma (MPM) is a cisplatin/pemetrexed (MTA) regimen; however, this is confronted by drug resistance. Proteotoxic stress in the endoplasmic reticulum (ER) is a hallmark of cancer and some rely on this stress signalling in response to cytotoxic chemotherapeutics. We hypothesise that ER stress and the adaptive unfolded protein response (UPR) play a role in chemotherapy resistance of MPM.

**Methods:**

In vitro three-dimensional (3D) and ex vivo organotypic culture were used to enrich a chemotherapy-resistant population and recapitulate an in vivo MPM microenvironment, respectively. Markers of ER stress, the UPR and apoptosis were assessed at mRNA and protein levels. Cell viability was determined based on acid phosphatase activity.

**Results:**

MPM cells with de novo and/or acquired chemotherapy resistance displayed low ER stress, which rendered the cells hypersensitive to agents that induce ER stress and alter the UPR. Bortezomib, an FDA-approved proteasome inhibitor, selectively impairs chemotherapy-resistant MPM cells by activating the PERK/eIF2α/ATF4-mediated UPR and augmenting apoptosis.

**Conclusions:**

We provide the first evidence for ER stress and the adaptive UPR signalling in chemotherapy resistance of MPM, which suggests that perturbation of the UPR by altering ER stress is a novel strategy to treat chemotherapy-refractory MPM.

## Introduction

Malignant pleural mesothelioma (MPM) is a relatively rare but extremely aggressive cancer originating from transformation of mesothelial cells of the pleura. The aetiology of MPM is closely associated with asbestos exposure.^1^ Albeit prohibition of commercial use of asbestos in early 1990s, the incidence of MPM worldwide is still increasing because of the long latency (30–60 years) of asbestos-associated carcinogenesis.^1^ Major histological subtypes of MPM include epithelioid, biphasic (or mixed) and sarcomatoid variants.^2^ The prognosis of patients with MPM is dismal, with a median survival time of 9–12 months only and a 5-year survival rate less than 5%.^3, 4^ No targeted therapies against MPM are available in the clinic, so current standard-of-care for patients with advanced MPM remains a dual chemotherapeutic regimen that combines cisplatin and pemetrexed (MTA).^5^ Clinical evidence indicates that this therapeutic regimen elicits only modest efficacy,^6^ rarely achieving durable clinical response because of drug resistance, de novo and/or acquired after initial therapeutic administration.^5, 7^ Little is known about the mechanism of chemotherapy resistance in MPM patients. As a consequence, no second-line treatment has been approved for MPM patients who fail the first-line chemotherapy.^8^

The endoplasmic reticulum (ER) plays a pivotal role in regulating proteostasis.^9^ Newly synthesised secretory proteins are first translocated into the lumen of the ER, where they are folded and assembled before being delivered to intra- and extracellular destinations where they acquire their function. This process is mediated by chaperone proteins resident in the ER lumen.^10^ When threatened by unsurmountable protein-folding demand, ER stress is initiated, which subsequently induces the unfolded protein response (UPR), an adaptive signalling pathway that evolved to alleviate proteotoxic stress placed on the ER and to restore ER homoeostasis.^11^ The UPR pathway coordinates cellular response to ER stress by integrating a complex signalling network, including the cascade mediated by protein kinase RNA-like endoplasmic reticulum kinase (PERK), a type I transmembrane protein resident on the ER. Upon activation, PERK phosphorylates the eukaryotic initiation factor eIF2α, leading to reduction in global protein synthesis but increased the expression of the activating transcription factor 4 (ATF4). ATF4 then transcriptionally alters its downstream effectors, e.g., the CCAAT/enhancer-binding protein homologous protein (CHOP). CHOP is involved in activation of apoptosis by blocking anti-apoptotic Bcl-2 family.^12, 13^

ER stress-induced activation of the UPR is an important mechanism of protein quality control in the ER that promotes cell survival.^14, 15^ However, irresolvable or persistent ER stress is detrimental, as it activates apoptosis.^16, 17^ Thus, inducing ER stress has emerged as a promising strategy for cancer therapy,^11, 18^ alone and combined with chemotherapy^19^ or targeted agents that inhibit oncogenic driver mutations.^20^ Prolonged disruption of the UPR can be achieved by pharmacological induction of ER stress. Bortezomib, an FDA-approved proteasome inhibitor, induces cell death by eliciting sustained ER stress.^21, 22^

In this study, we reported that deregulated UPR signalling sensitises chemotherapy-resistant MPM cells to agents that induce ER stress. We showed that MPM cells resistant to first-line cisplatin/MTA chemotherapy display low basal level of ER stress and that bortezomib selectively impairs these cells by eliciting differential UPR, elevating the response to ER stress and augmenting apoptotic cell death. Mechanistically, hyperactivation of the PERK/ATF4/ CHOP pathway is important for bortezomib-induced UPR and apoptosis. Our study thus reveals an important role for UPR signalling in chemotherapy resistance of MPM, and offers a new rationale by altering ER stress to treat patients with chemotherapy-resistant MPM.

## Materials and methods

### Cell culture and reagents

The MPM cell line H28 and a normal mesothelial cell line (Met-5A) were obtained from ATCC (American Type Culture Collection, Manassas, VA, USA). MPM cell lines MESO-1 (ACC-MESO-1) and MESO-4 (ACC-MESO-4) were purchased from RIKEN Cell Bank (Ibaraki, Japan) and described previously.^23^ MPM cell lines (MSTO-211H and JL-1) were obtained from DSMZ (German Collection of Microorganisms and Cell Cultures, Brunswick, Germany). Cells were cultured in RPMI-1640 medium or Medium 199 (Cat. #8758 and #4540; Sigma-Aldrich, St. Louis, MO, USA) supplemented with 10% foetal bovine serum/FBS (Cat. #10270-106; Life Technologies, Grand Island, NY, USA) and 1% penicillin/streptomycin solution (Cat. #P0781, Sigma-Aldrich, St. Louis, MO, USA) at 37 °C with 95% air/5% CO_2_ in a humid condition. The authenticity of the cell lines was verified by DNA fingerprinting (Microsynth, Bern, Switzerland) and they were tested to be free from mycoplasma contamination. Three-dimensional culture of tumourspheres was performed as previously described.^24^ In brief, single cells suspended in MammoCult™ Human Medium (Cat. #05620; STEMCELL Technologies, Canada) were plated (1000 cells/ml) in ultra-low attachment plates (Cat. #3471; Corning Incorporated, Corning, NY, USA) and cultured for 5–7 days. To optimise sphere growth, cell culture medium was replenished every 3 days. Chemotherapeutic agents cisplatin and pemetrexed (commercial name “ALIMTA”; Cat #VL7640) were purchased from Sandoz and Eli Lilly (Suisse) S.A. (Vernier/Geneva, Switzerland), respectively. HA15 and bortezomib were obtained from Selleckchem (Cat. #S8299, and #S1013; Houston, USA) and Thapsigargin from Tocris (Cat. #1138; Bristol, UK).

### Cell viability and clonogenic survival assay

2D- and 3D-cultured cells (2500 cells/well) were seeded in 96-well plates. After 24 h, cells were treated with various drugs for 72 h, unless otherwise indicated. Cell viability was determined by acid phosphatase (APH) assay as described.^25^ The efficacy of drugs on cell growth was normalised to untreated control. Each data point was generated in triplicate and each experiment was repeated twice. Unless otherwise stated, a representative result is presented. Best-fit curve was generated in GraphPad Prism [(log (inhibitor) vs response (-variable slope four parameters)]. Error bars are mean ± s.d.

Clonogenic assay was performed as described.^26^ In brief, exponentially grown MPM cells were seeded in six-well plates at a clonal density of 5000 cells/well and treated with various reagents for the indicated time period. After 7–10 days depending on growth rate, the resulting colonies were stained with crystal violet (0.5% dissolved in 25% methanol). Growth curve was generated by eluting crystal violet staining with 10% acetic acid and measuring absorbance at 590 nm.

### Quantitative real-time PCR (qRT-PCR)

Total RNA was isolated and purified with RNeasy Mini Kit (Cat. #74106, Qiagen, Germany). Complementary DNA (cDNA) was synthesised by the high capacity cDNA reverse transcription kit (Cat. # 4368814, Applied Biosystems, Foster City, CA, USA) according to the manufacturer’s instructions and qPCR analyses were performed in triplicate on a 7500 Fast Real-Time PCR System (Applied Biosystems) with commercially available TaqMan “Assay on Demand” primer/probes (Supplementary Table S2). The expression level of each target gene was normalised against *GAPDH* and compared among different groups by the ^ΔΔ^CT method. Baseline and threshold for Ct calculation were set automatically with the 7500 software v2.06.

### Immunoblotting and immunohistochemistry

Cell lysates were prepared and western blot analysis was performed as described,^27^ with the exception that protease inhibitors (Cat. #78440; Thermo Fisher Scientific, MA, USA) were included in lysis buffer. In brief, equal amounts of protein lysates (10–25 μg/lane) were resolved by SDS-PAGE (Cat. #4561033; Bio-Rad Laboratories, Hercules, CA, USA) and transferred onto nitrocellulose membranes (Cat. #170-4158; Bio-Rad). Membranes were then blocked in blocking buffer (Cat. #927-4000; Li-COR Biosciences, Bad Homburg, Germany) for 1 h at room temperature and incubated with appropriate primary antibodies overnight at 4 °C (Supplementary Table S3). IRDye 680LT-conjugated goat anti-mouse IgG (Cat. #926-68020) or IRDye 800CW-conjugated goat anti-rabbit IgG (Cat. #926-32211) from Li-COR Biosciences were used at 1:10000 dilutions. Finally, signals of membrane-bound secondary antibodies were imaged using the Odyssey Infrared Imaging System (Li-COR Biosciences).

Surgically removed syngeneic and xenograft tumours were formalin-fixed and paraffin-embedded (FFPE) and stained with haematoxylin and eosin (H&E) using a standard protocol. FFPE tissue blocks were sectioned at 4μm, deparaffinised and rehydrated. Subsequent immunohistochemical staining with appropriate antibodies (Supplementary Table S3) was performed with the automated system BOND RX (Leica Biosystems, Newcastle, UK). Visualisation was performed using the Bond Polymer Refine Detection kit (Leica Biosystems) as instructed by the manufacturer. Images were acquired and processed using Adobe Photoshop CS6 v13 (Adobe Systems Incorporated).

### Patient-derived xenografts (PDX) model and ex vivo organotypic tissue culture

NSG mice (NOD/SCIDγ^-/-^) were anesthetised by i.p. injection of 50 μl narcotics mix (0.5 mg/kg dormitor/5 mg/kg dormicum/0.05 mg/kg fentanyl) and shaved (left and right back flank). Surgery was immediately started when reflexes disappear. After disinfection with 70% ethanol, a subcutaneous pocket was created by two small incisions over the left and right back flank. Fresh MPM tissues, surgically resected at the Division of Thoracic Surgery, Bern University Hospital, and subsequently dissected by a pathologist at the Institute of Pathology, University of Bern, were cut into small pieces (5 μm x 5 μm) and implanted in the pocket. Finally, the incisions were closed by surgical clips and each mouse was injected (s.c.) with 100μl antidot mix (1.1 mg/kg Alzane/0.45 mg/kg anexate/0.075 mg/kg temgesic). Tumours of 15 mm in diameter were harvested for further analysis and mice were euthanised.^28^ All mouse studies were conducted in accordance with Institutional Animal Care and Ethical Committee-approved animal guidelines and protocols.

For ex vivo organotypic culture, freshly explanted PDX tumours were processed immediately. In brief, explanted tumour tissues were soaked in ice-cold sterile PBS with antibiotic/antimycotic, mounted on agarose and cut into slices (300–500 μM) by a Vibratome VT1200 (Leica Microsystems).^29^ Tissue slices were cultured in ultra-low attachment plates (Cat. #3471; Corning Incorporated, Corning, NY, USA) in DMEM supplemented with 20% FBS (Cat. #10270-106; Life Technologies, Grand Island, NY, USA) and 1% penicillin/streptomycin solution (Cat. #P0781, Sigma-Aldrich, St. Louis, MO, USA) at 37 °C and 95% air/5% CO_2_. After 12 h, drugs were added and treatment lasted for up to 72 h. Slices collected at baseline time (T0) and after treatment were snap-frozen for qPCR and in 10% formalin for immunohistochemical staining.

### Apoptosis assays

MPM cells were treated for 48 h with vehicle control or the indicated drugs. After treatment, cells in the supernatant and adherent to plates were collected, washed with PBS and pooled before suspended in 500 µl binding buffer and stained with the Annexin V Apoptosis Detection Kit -FITC (Cat. #88-8005; Thermo Fisher Scientific, MA, USA) according to the manufacturer’s instructions. Flow cytometry analysis was performed on a BD Biosciences LSRII flow cytometer. Three independent experiments were performed.

For the assay using apoptosis antibody array, protein lysates (200 μg) prepared from bortezomib- and vehicle-treated MPM cells were analysed by a Human Apoptosis Array Kit (Cat. # ARY009, R&D Systems, Minneapolis, MN, USA) according to the manufacturer’s protocol.

### siRNA knockdown

Knockdown of CHOP was achieved by specific duplex siRNAs (50nmol/L) purchased from Origene Technologies (Cat. #SR319903). Transfection of siRNAs was performed with SiTran1.0 (Cat. #TT300001, Origene Technologies, MD, USA) according to the manufacturer’s instructions.

### Statistical analysis

Statistical analyses were performed using GraphPad Prism 6.03 (GraphPad Software Inc., http://www.graphpad.com/welcome.htm) unless otherwise indicated. In all studies, data represent biological replicates (n) and are depicted as mean values ± s.d. as indicated in the figure legends. Comparison of mean values was conducted with unpaired, two-tailed Student’s *t*-test or one-way ANOVA with Donnett’s *post hoc* test as indicated in the figure legends. In all analyses, *P* values less than 0.05 were considered statistically significant.

## Results

### Characterisation of MPM cells resistant to standard chemotherapy

In vitro 3D culture more accurately recapitulates in vivo tumour microenvironment and therapeutic response that ultimately occurs in patients.^30, 31^ To test if 3D culture can be used to enrich chemotherapy-resistant population in MPM cells, we compared MPM cells propagated under 3D culture conditions (thereafter referred to as 3D cells) and parental tumour cells grown in monolayer (referred to as 2D cells) for their response to chemotherapy. 3D and 2D cells were treated with standard chemotherapeutic regimen and cell viability was determined 72 h after the treatment. Contrasted to parental H28, MESO-1 and MESO-4 cells (2D) where cisplatin and pemetrexed/MTA exerted dosage-dependent inhibition of tumour cell growth, the 3D populations derived from the three well-established MPM cell lines showed unanimously reduced sensitivity to cisplatin and MTA, regardless of whether they were used alone (Supplementary Figure S1A) or in combination (Supplementary Figure S1B). These results indicate that MPM cells propagated by in vitro 3D culture are resistant to standard chemotherapy.

### Chemotherapy-resistant MPM cells display low ER stress and the adaptive UPR signalling

**Fig. 1:**
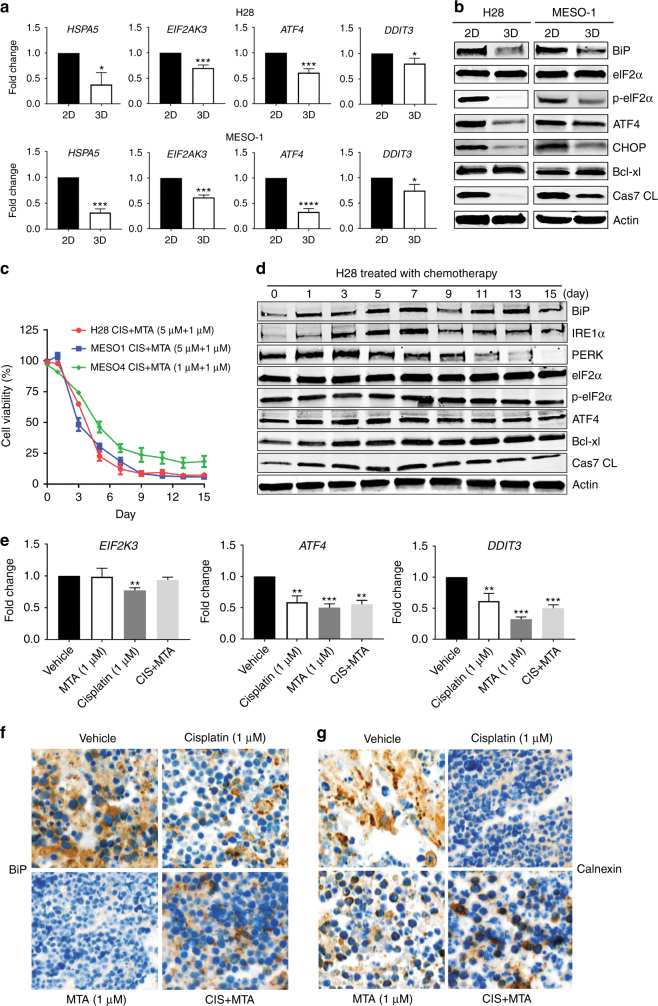
Chemotherapy-resistant MPM cells display low ER stress and the adaptive UPR signalling. **a** qRT-PCR of 3D cells and parental cells (2D) for genes involved in the UPR (*HSPA5*, *EIF2AK3*, *ATF4* and *DDIT3*). Data are presented as mean ± s.d. (*n* = 3). *P < 0.05, ***P < 0.001 and ****P < 0.0001 by unpaired two-sided t-test. **b** Immunoblots of 3D and parental cells for UPR-related proteins and apoptotic markers. **c** Viability of MPM cells after treated with cisplatin/MTA for the indicated time periods. Data are presented as mean ± s.d. (*n* = 3). **d** Immunoblots for UPR-related proteins and apoptotic markers in H28 cells that were treated as in (**c**). **e** qRT-PCR of BE454 xenograft tumours cultured ex vivo and treated with cisplatin/MTA for 72 h. Data are presented as mean ± s.d. (*n* = 3). ** *P* < 0.01 and ***P < 0.001 by unpaired two-sided t-test. (**f** and **g**) IHC for BiP (**f**) and Calnexin (**g**) in BE454 PDX tumours that were cultured and treated as in (**e**). Original overall magnification, ×400

ER stress and the adaptive UPR have emerged as a key mechanism of chemotherapy resistance.^19^ We determined if the UPR has a role in response of MPM cells to chemotherapy. Quantitative real-time PCR (qRT-PCR) showed significant downregulation of a panel of the UPR-related genes, including *HSPA5* (BiP), *EIF2AK3* (PERK), *ATF4 (*ATF4*)* and *DDIT3* (CHOP), in 3D cells compared to 2D counterparts (Fig. 1a). Western blots confirmed that BiP, ATF4, CHOP and phosphorylated eIF2α (p-eIF2α), key markers of UPR activity, were dramatically reduced in 3D cells versus the parental H28 and MESO-1 cells (Fig. 1b). Notably, the decrease in UPR activity correlated with a reduced apoptotic index in 3D cells, marked by elevated anti-apoptotic Bcl-xl and reduced cleaved caspase 7, a marker of apoptosis (Fig. 1b).

To determine how chemotherapy modulates UPR signalling, H28, MESO-1 and MESO-4 cells were chronically treated with cisplatin and MTA. Cisplatin/MTA exerted time-dependent growth inhibition ( < 9 days), but failed to do so in prolonged treatment ( > 9 days), indicating emergence of chemotherapy resistance (Fig. 1c). Notably, despite temporal increase, the protein level of IRE1α, PERK, p-eIF2α and ATF4 in H28 cells was steadily suppressed by cisplatin/MTA, and this suppression correlated with decreased sensitivity to chemotherapy in the treated cells. In particular, the PERK/p-eIF2α/ATF4 pathway was substantially inactivated by cisplatin/MTA, as PERK and p-eIF2α, and, to a lesser extent, ATF4, were markedly downregulated after treated for 15 days compared to those at d0 (Fig. 1d). Consistently, cisplatin/MTA induced Bcl-xl and reduced cleaved caspase 7 in H28 cells (Fig. 1d).

To interrogate the in vitro results, xenograft tumours derived from a MPM patient (BE454; Supplementary Table S1) were subjected to ex vivo organotypic culture^28, 29^ and treatment. Cisplatin/MTA (72 h) substantially decreased the expression of *ATF4* and *DDIT3* (Fig. 1e) and of BiP and calnexin, protein chaperons and markers of ER stress (Fig. 1f, g). These results indicate that chemotherapy resistance in MPM is associated with repressed ER stress and low apoptotic index.

### Deregulated UPR activity sensitises chemotherapy-resistant MPM cells to ER stress

**Fig. 2:**
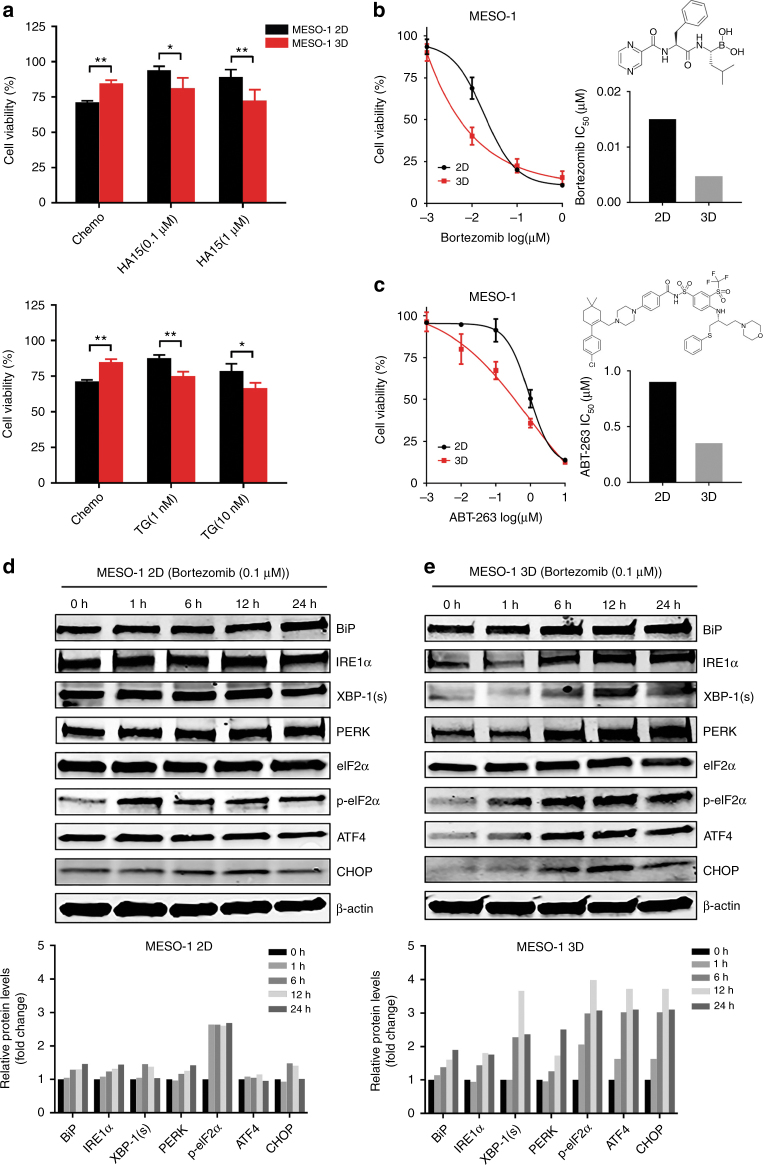
Chemotherapy-resistant MPM cells are hypersensitive to ER stress. **a** Viability of 2D and 3D cells (MESO-1) after treated with chemo (1 μM cisplatin + 1 μM MTA) or the indicated agents for 72 h. Data are presented as mean ± s.d. (*n* = 3). *P < 0.05, ** *P* < 0.01, ***P < 0.001 by unpaired two-sided t-test. ns, not significant. **b**, **c** Viability and IC_50_ of 2D and 3D cells after treated with bortezomib or ABT-263 for 72 h. Data are presented as mean ± s.d. (*n* = 3). **d**, **e** Immunoblots of 2D and 3D cells after treated with bortezomib (0.1 μM) for the indicated time periods. Quantification of the protein levels is shown at the bottom

To determine if deregulated UPR signalling renders chemotherapy-resistant MPM cells vulnerable to ER stress, 3D and 2D of MESO-1 cells were treated with HA15, thapsigargin and bortezomib, agents that induce ER stress.^20, 32, 33^ HA15 and thapsigargin lowered MESO-1 viability in a dose-dependent manner, but were more deleterious for 3D than 2D cells (Fig. 2a). Similarly, bortezomib and ABT-263, FDA-approved inhibitors of proteasome and anti-apoptotic Bcl-2 family, respectively, impaired the viability of 3D cells to a much greater extent than of parental MESO-1 cells (Fig. 2b, c), supporting the notion that chemotherapy-resistant MPM cells are more resistant to apoptosis (Fig. 1d).

**Fig. 3:**
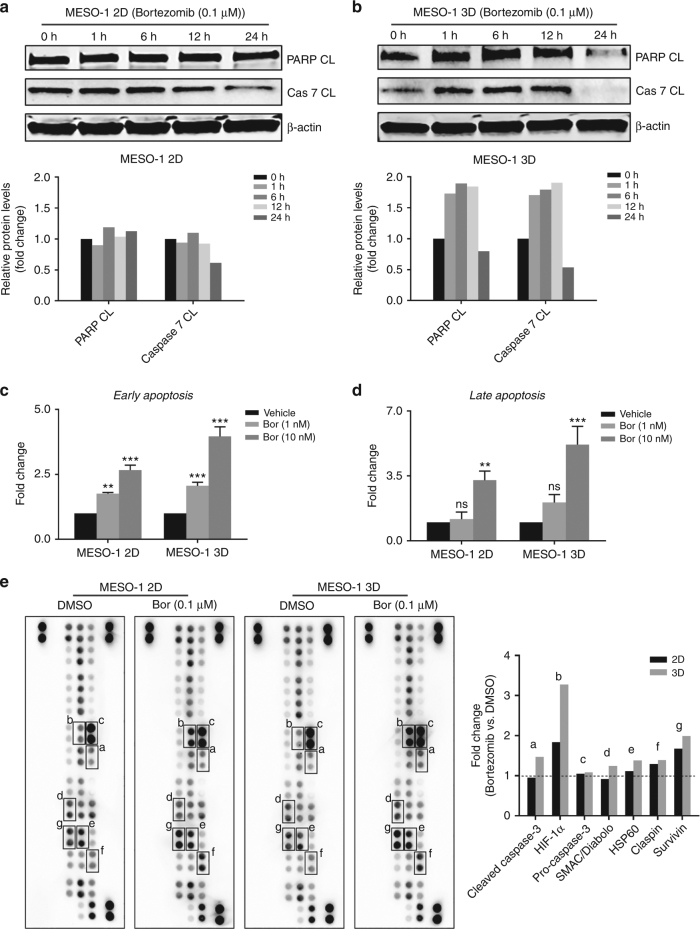
Bortezomib sensitises chemotherapy-resistant MPM cells to apoptosis by eliciting an elevated UPR. **a**, **b** Immunoblots for apoptotic markers in 2D and 3D cells (MESO-1) after treated with bortezomib (0.1 μM) for indicated time periods. Quantification of the protein level was shown underneath. **c**, **d** FACS analysis for apoptotic cell death in 2D and 3D cells (MESO-1) after treated with bortezomib for 48 h. **e** Antibody-based array assay for apoptosis-related proteins in 2D and 3D cells treated with bortezomib (0.1 μM) or vehicle (DMSO) for 6 h. The relative level (bortezomib vs. vehicle) of proteins that were perturbed by bortezomib is shown to the right. The protein level in vehicle-treated cells was set as 1

We then determined if the efficacy difference on 2D and 3D cells was due to differential UPR to bortezomib-induced ER stress. Bortezomib (0.1 μM) elicited acute and time-dependent increase in BiP, IRE1α XBP-1(s), PERK, p-eIF2α, ATF4 and CHOP in 3D cells, but only marginally affected the expression of these proteins in the parental MESO-1 cells (Fig. 2d, e). Importantly, in 3D cells bortezomib (0.1μM) markedly increased the level of apoptotic markers cleaved poly(ADP-ribose) polymerase (PARP CL) and Caspase-7 (Cas-7 CL) that, however, were only mildly affected in the parental cells (Fig. 3a, b). Fluorescence activated cell sorting (FACS) analysis revealed that bortezomib at low doses (1, 10 nM) induced significantly greater apoptosis in 3D than in 2D cells (Fig. 3c, d). Similar results were obtained from an apoptosis array assay, in which bortezomib treatment (6 h) induced the expression of cleaved caspase-3, a critical executioner of apoptosis, in 3D cells but not in 2D cells: the relative ratio of cleaved caspase-3 in bortezomib- versus DMSO-treated cells was 1.46 in 3D cells and 0.95 in 2D cells (Fig. 3e). Consistently, bortezomib promoted the expression of pro-apoptotic SMAC^34^ and, to a lesser extent, of Hsp60,^35^ increasing the level of the two proteins by 38% and 24% (compared to DMSO) in 3D cells (Fig. 3e). In contrast, bortezomib repressed SMAC and only slightly increased Hsp60 (11%) in 2D cells (Fig. 3e). We also observed bortezomib-dependent increase of pro-survival Claspin^36^ and Survivin,^37^ but to similar extent in 2D and 3D cells (Fig. 3e). Interestingly, HIF-1α, a key transcription factor activated by hypoxia,^38^ was dramatically increased by bortezomib, more robustly in 3D than 2D cells (Fig. 3e). HIF-1α can evoke both pro-apoptotic and pro-survival signalling, depending on contextual stimuli.^39^ Future studies are required to elucidate the link between bortezomib-induced HIF-1α, induction of apoptosis and chemotherapy response in MPM cells.

Together, these results demonstrate that bortezomib exerts selective cytotoxicity in chemotherapy-resistant MPM cells. This efficacy difference is associated with differential adaption of parental (2D) and 3D cells in response to bortezomib-elicited ER stress, resulting in elevated UPR activation and augmented apoptotic cell death in chemotherapy-refractory MPM cells.

### Bortezomib promotes cisplatin/MTA efficacy in chemotherapy-resistant MPM cells

**Fig. 4:**
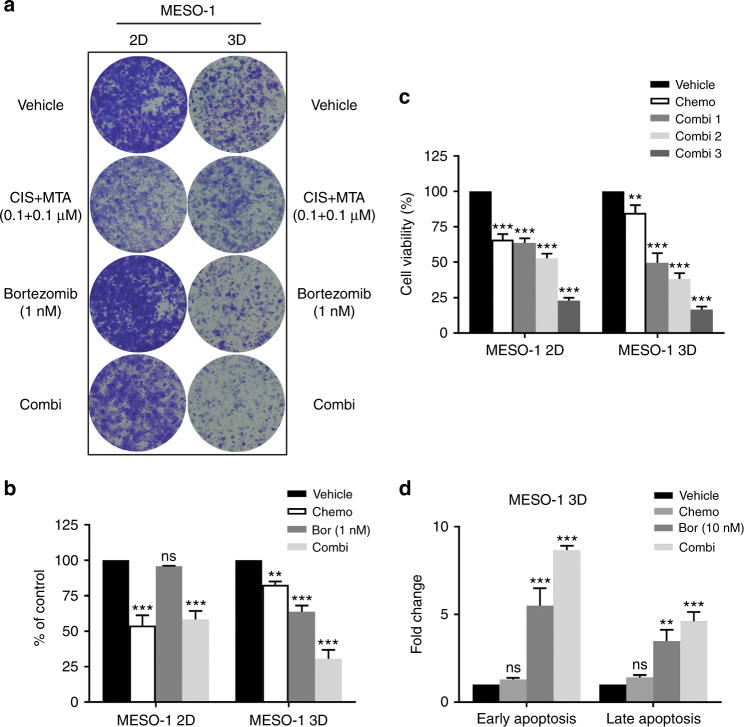
Bortezomib promotes cisplatin/MTA efficacy in chemotherapy-resistant MPM cells. **a**, **b** Clonogenic assay of 2D and 3D MESO-1 cells after treated with the indicated drugs. Colonies were stained (**a**) and quantified (**b**) after 7 days. Data are presented as mean ± s.d. (*n* = 3). **P < 0.01, ***P < 0.001 by unpaired two-sided t-test. ns, not significant. **c** Viability of 2D and 3D cells after 72 h treatment with chemotherapy (1 μM cisplatin + 1 μM MTA) alone or combined with bortezomib (1, 10, 100 nM). Data are shown as mean ± s.d. (*n* = 3). **P < 0.01, ***P < 0.001 by unpaired two-sided t-test. **d** FACS-based apoptotic assay of 3D cells after treated for 48 h with chemotherapy (1 μM cisplatin + 1 μM MTA), bortezomib (10 nM) or the combination

Given that bortezomib preferentially impairs chemotherapy-resistant MPM cells, we next determined if bortezomib could restore chemotherapy-resistant MPM cells to standard therapy. First, supporting the results of viability analysis (Supplementary Figure S1), clonogenic assay showed that 3D cells were highly resistant to cisplatin/MTA regimen (0.1 μM cisplatin and 0.1 μM MTA) but hypersensitive to bortezomib relative to 2D cells (Fig. 4a, b). Concurrent treatment with bortezomib enhanced the inhibitory effect of cisplatin/MTA in 3D cells, outperforming single agents alone; in contrast, this combinatorial effect was not observed in the parental MESO-1 cells (Fig. 4a, b). Second, viability assay confirmed that bortezomib (10 nM, 100 nM and 1 μM) reduced viability of 3D cells in a dose-dependent manner when combined with cisplatin (1 μM)/MTA (1 μM) regimen (Fig. 4c). Although bortezomib also promoted cisplatin/MTA efficacy in parental MESO-1 cells, the combined effect was less robust than that in 3D cells (Fig. 4c). Finally, FACS analysis confirmed that combined bortezomib and cisplatin/MTA triggered significantly greater apoptosis in chemotherapy-resistant MPM cells than monotherapy of chemotherapy or bortezomib (Fig. 4d).

### MPM cells with low ER stress are intrinsically resistant to chemotherapy but hypersensitive to bortezomib

**Fig. 5:**
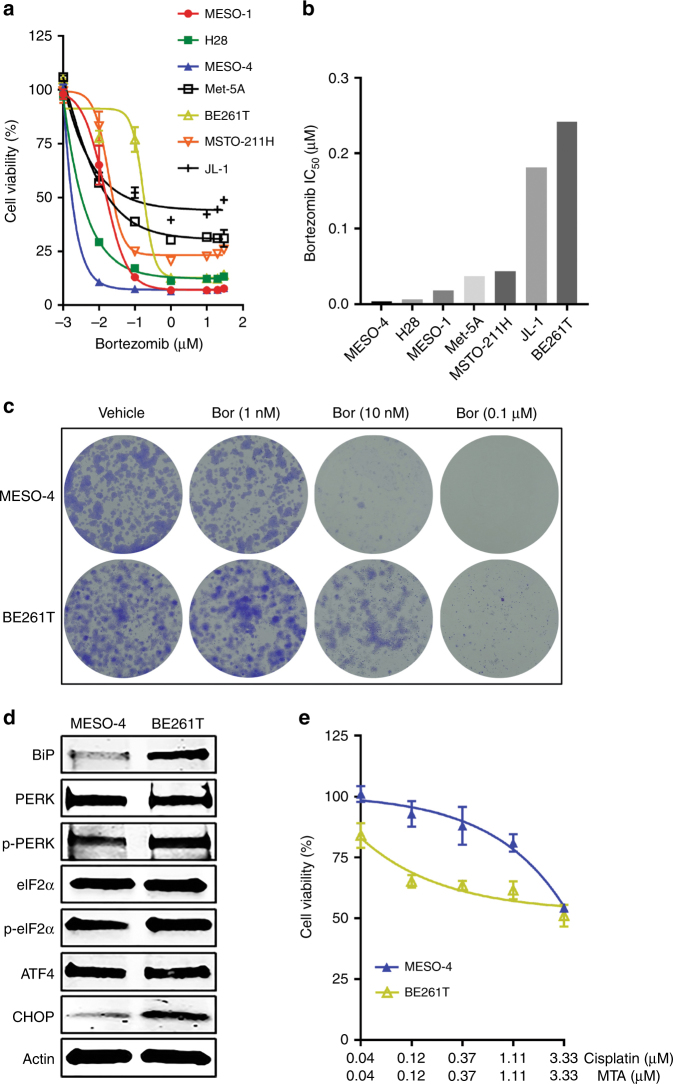
MPM cells with low ER stress/UPR signalling are intrinsically resistant to chemotherapy. **a**, **b** Viability (**a**) and IC_50_ (**b**) of MPM lines and normal mesothelial cells (Met-5A) after treatment with bortezomib for 72 h. Data are presented as mean ± s.d. (*n* = 3). **c** Clonogenic assay of MESO-4 and BE261T cells treated with the indicated doses of bortezomib. **d** Immunoblots of MESO-4 and BE261T cells for proteins involved in the UPR and apoptosis. **e** Viability of MESO-4 and BE261T cells after treated with cisplatin/MTA for 72 h

Next, we tested if ER stress and the adaptive UPR play a role in intrinsic chemotherapy resistance in MPM. Drug sensitivity profiling of a panel of MPM cell lines (MESO-1, H28, MESO-4, MSTO-211H, JL-1), patient-derived primary MPM cells (BE261T) (Supplementary Table S1) and a non-transformed normal mesothelial cell line (Met-5A) revealed that the cells varied in their response to bortezomib, with MESO-4 showing the highest sensitivity and BE261T the greatest resistance (Fig. 5a, b). Clonogenic assay confirmed that MESO-4 cells were more sensitive to bortezomib than BE261T cells (Fig. 5c). Notably, MESO-4 cells that were susceptible to bortezomib displayed low ER stress and the adaptive UPR activity, manifested by dramatically lower levels of BIP, p-PERK, p-eIF2a and CHOP than those in BE261T cells (Fig. 5d). Importantly, MESO-4 cells were intrinsically more resistant to cisplatin/MTA than BE261T cells (Fig. 5e). Hence, chemotherapy resistance correlates with low ER stress and UPR signalling, which renders chemotherapy-resistant MPM cells selectively susceptible to agents that induce ER stress, regardless of whether the resistance is due to an intrinsic or induced mechanism. These results have important clinical implications, which suggests that stratification of patients with newly diagnosed MPMs according to the magnitude of proteotoxic ER stress and the adaptive UPR signalling may predict those who will likely benefit from bortezomib-based therapy.

### PERK/ATF4/CHOP pathway plays a pivotal role in bortezomib-elicited UPR and apoptosis

**Fig. 6:**
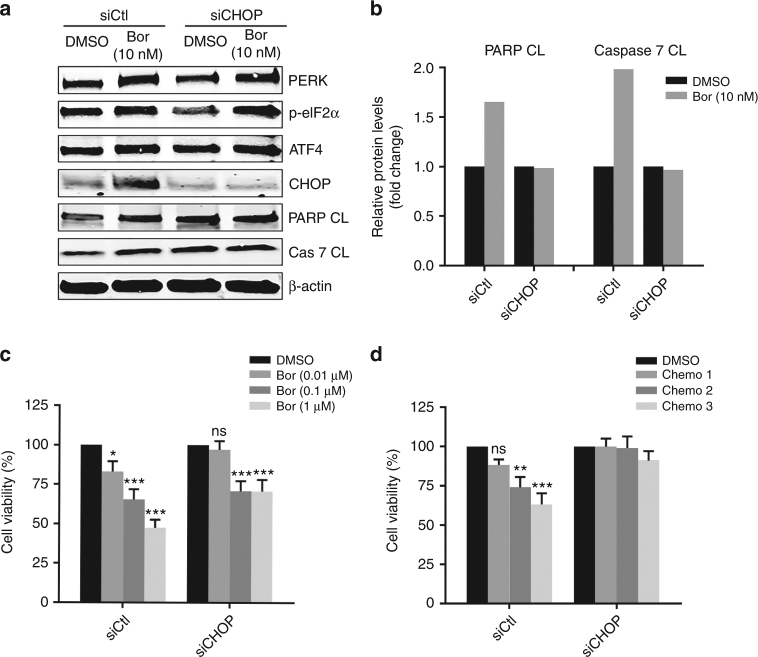
PERK/ATF4/CHOP pathway is functionally important for bortezomib-induced UPR and apoptosis. **a**, **b** MESO-1 cells transfected with control siRNA (siCtl) or siRNA against CHOP (siCHOP) were treated for 24 h with bortezomib and analysed by immunoblotting (**a**). Quantification of apoptotic markers (PARP CL and Caspase 7 CL) is shown in (**b**). **c**, **d** MESO-1 cells transfected with control or CHOP siRNA were treated with the indicated doses of bortezomib or cisplatin/MTA (chemo 1: 0.5 μM/1 μM; chemo 2: 1 μM/1 μM; chemo 3: 5 μM/1 μM). Cell viability was measured 24 h (bortezomib) or 48 h (cisplatin/MTA) after treatment. Data are shown as mean ± s.d. (*n* = 3). *P < 0.05, **P < 0.01, ***P < 0.001 by unpaired two-sided t-test

UPR signalling transmitted by the PERK/ATF4/CHOP pathway was markedly repressed by cisplatin/MTA (Fig. 1d, e) but significantly activated by bortezomib (Fig. 2e), which prompted us to investigate if this pathway is functionally important for bortezomib-elicited UPR and apoptosis. As expected, RNAi-based depletion of *DDIT3* (encoding CHOP) precluded bortezomib-induced expression of CHOP, but not of those upstream of CHOP, such as PERK, p-eIF2α and ATF4 that were equally induced by bortezomib in MESO-1 cells with or without CHOP depletion (Fig. 6a). Importantly, bortezomib treatment (24 h) failed to induce cleaved PARP and caspase 7 in CHOP-depleted MESO-1 cells, whereas they were substantially increased by bortezomib in MESO-1 transfected with control siRNA (Fig. 6b). To be noted, bortezomib inhibited MESO-1 viability in a dose-dependent manner, but CHOP depletion significantly compromised this inhibitory effect (Fig. 6c). Last but not least, cisplatin/MTA exerted dose-dependent effectiveness in MESO-1 cells; however, CHOP knockdown abolished the response to cisplatin/MTA (Fig. 6d). These results demonstrate that integrity of the PERK/ATF4/CHOP pathway is indispensable for bortezomib-induced UPR and apoptosis in MPM cells.

In summary, we demonstrated here that ER stress and the adaptive UPR pathway play a fundamental role in chemotherapy resistance of MPM, and that bortezomib, an FDA-approved proteasome inhibitor, is selectively toxic for chemotherapy-resistant MPM cells by evoking an elevated UPR. Our findings suggest that MPM patients with recurrent disease after first-line treatment or tumours intrinsically refractory to chemotherapy may benefit from bortezomib-based treatment.

## Discussion

Clinical treatment for MPM is limited.^40^ Therapeutic options for patients with early stage MPM, accounting for 10–15% of all MPM cases,^41^ include surgical resection and multimodal therapies that combine chemotherapy or radiation therapy with surgery in different orders.^42, 43^ For patients with advanced-stage, unresectable disease, currently approved first-line therapy is a dual chemotherapy regimen with cisplatin plus pemetrexed that modestly improved the median survival time from 9 to 12 months.^6^ However, MPM is largely unresponsive, with only 40% of MPM patients with MPM show a clinical response to this treatment,^6, 44^ and patients who initially respond to therapy eventually develop resistance.^45^ Currently, there are no second-line therapies have been clinically approved for therapy-refractory or relapsed MPM.^8^ It is therefore paramount to identify and develop new therapeutic approaches to improve clinical outcome of MPM patients. A better understanding of the molecular underpinnings underlying MPM resistance to existing therapies may hold the promise to meet this unmet need.

In this study, we addressed one of the major clinical challenges for MPM: resistance to front-line cisplatin/MTA chemotherapy.^46^ Using in vitro and ex vivo models, we provided the first evidence for a role of ER stress and the adaptive UPR signalling pathway in chemotherapy resistance of MPM, regardless of the resistance mechanism (intrinsic or acquired). We further showed that deregulated UPR activity in chemotherapy-refractory MPM cells renders these cells hypersensitive to agents that induce ER stress and augment the UPR signalling. These findings thus offer a rationale by perturbing ER stress and the adaptive UPR to treat patients whose MPM tumours have progressed through, become resistant to and/or relapsed after first-line chemotherapy.

A vast majority of patients present with advanced-stage, unresectable MPM at diagnosis.^45^ The only approved treatment for these patients is the cisplatin/MTA combination regimen. More disappointingly, patients that initially respond to this therapy inevitably develop resistance.^45^ A key finding of our present study is that MPM cells resistant to cisplatin/MTA therapy display low ER stress and the adaptive UPR signalling, regardless of whether the resistance is due to an acquired or intrinsic mechanism. This finding is mostly consistent with earlier studies showing that chemotherapy-resistant stem cells in normal intestine or colon cancer are low in ER stress compared to their differentiated counterpart.^19, 47^ Although beyond the scope of this study, it will be highly pertinent in the future to elucidate the nature of chemotherapy-resistant MPM cells, in particular if they are equivalent to cancer stem cells in MPM.

We further demonstrated that deregulated UPR signalling in chemotherapy-resistant MPM cells licences an increased sensitivity of these cells to agents that induce ER stress and perturb proteostatic signalling pathways. Indeed, several ER stress inducers, including thapsigargin, HA15 and the FDA-approved proteasome inhibitor bortezomib, selectively impaired chemotherapy-resistant MPM cells. We showed that bortezomib exerts the efficacy by eliciting differential UPR, elevating the response to ER stress and augmenting apoptotic cell death in chemotherapy-resistant MPM cells. Compared to bortezomib and chemotherapy alone, concurrent treatment triggered even greater UPR and apoptotic response in chemotherapy-resistant MPM cells, indicating that bortezomib further promotes the efficacy of cisplatin/MTA regimen. Mechanistically, we showed that the UPR mediated by PERK/ATF4/CHOP pathway plays an important role in bortezomib-elicited apoptotic cell death, as CHOP depletion attenuated the efficacy of bortezomib. These results are in agreement with those reported from colon and brain cancer, where ER stress-induced activation of the UPR subverts chemotherapy resistance.^47–49^

Preclinical studies have unveiled a promising efficacy for bortezomib in a variety of tumours, including MPM.^50, 51^ However, clinical trials with bortezomib, alone or combined with chemotherapeutics, have led to dismal results in unselected MPM patients.^52–54^ Given our findings that bortezomib preferentially impairs chemotherapy-resistant MPM cells and that combined bortezomib with chemotherapy only mildly affects drug naïve parental MPM cells, future clinical investigations with bortezomib might need centre on (1) MPM patients with relapsed disease after initial chemotherapy; and (2) patients with newly diagnosed MPMs that are intrinsically resistant to chemotherapy.

Collectively, we report in this study that ER stress and the adaptive UPR play an important role in chemotherapy resistance of MPM, and that deregulated UPR activity in chemotherapy-resistant MPM cells sensitises the cells to agents that induce ER stress and alter the UPR. Bortezomib selectively impairs chemotherapy-resistant MPM cells by inducing differential UPR, leading to elevated response to ER stress and apoptotic cell death. Mechanistically, UPR signalling mediated by PERK/ATF4/CHOP pathway is indispensable for bortezomib-elicited apoptosis. Our study suggests that perturbation of the UPR signalling by altering ER stress may be a novel strategy to treat patients with chemotherapy-resistant MPM, either tumours recurrent from first-line treatment or those naïve to treatment but intrinsically refractory to chemotherapy.

## Electronic supplementary material

Supplementary material
