# Supplementary material for: Increased sensitivity to apoptosis upon endoplasmic reticulum stress-induced activation of the unfolded protein response in chemotherapy-resistant malignant pleural mesothelioma
Source: Br J Cancer. 2018 Jun 20;119(1):65–75. doi: 10.1038/s41416-018-0145-3 (PMC6035279; doi:10.1038/s41416-018-0145-3)
Supplement: Supplementary file 1 — Supplementary material [file 41416_2018_145_MOESM1_ESM.doc]

**SUPPLEMENTARY MATERIAL**

**
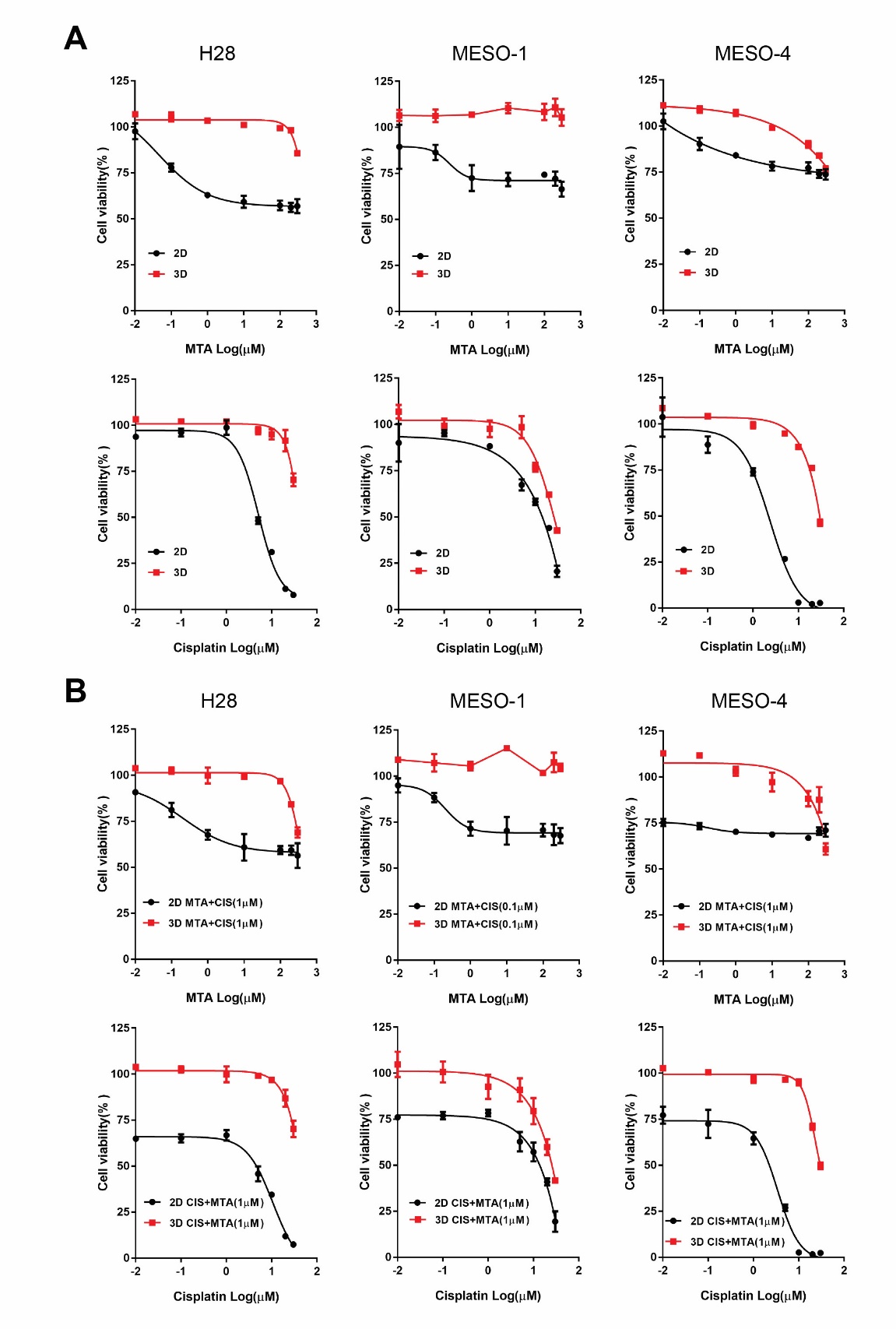
**

**Supplementary Figure S1. *In vitro* 3D culture enriches chemotherapy-resistant MPM cells**

(**A** and **B**) H28, MESO-1 and MESO-4 cells cultured in 2D or 3D were dissociated, seeded and treated with cisplatin and pemetrexed/MTA, alone (A) or in combination (B). Cell viability was determined 72 h after treatment. Data are presented as mean ± s.d. (n=3).

**Supplementary** Table S1. Characteristics of patient samples used in this study.

| **#** | **Age** | **Gender** | **Tumour** | **Histology** | **Therapy** | TNM Stage* |
| --- | --- | --- | --- | --- | --- | --- |
| BE261 | 67 | M | MPM |  | Cisplatin/MTA | ypT1b pN0 cM0 |
| BE454 | 53 | M | MPM |  | Cisplatin/MTA | ypT3 pN0 cM0 |

* Under TNM (7th edition, 2009)

**Supplementary Table S2**. Sequences of primers used in qRT-PCR

| **Gene** | **Species** | **Primer** | **Company** |
| --- | --- | --- | --- |
| *HSPA5* | human | Hs00607129_gH | ThermoFisher |
| *EIF2AK3* | human | Hs00984005_m1 | ThermoFisher |
| *ATF4* | human | Hs00909569_g1 | ThermoFisher |
| *DDIT3* | human | Hs00358796_g1 | ThermoFisher |

**Supplementary Table S3**. Antibodies used in this study

| **Antibody** (for WB) | **Source** | **Company** | **Cat. #** |
| --- | --- | --- | --- |
| BiP | Rabbit mAb | CST | 3183S |
| IRE1α | Rabbit mAb | CST | 3294S |
| PERK | Rabbit mAb | CST | 5683S |
| eIF2α | Rabbit mAb | CST | 5324S |
| Phospho-eIF2α(Ser 51) | Rabbit mAb | CST | 3398S |
| ATF4 | Rabbit mAb | CST | 11815S |
| Cleaved-caspase 7 | Rabbit mAb | CST | 8438S |
| BCL-xl | Rabbit mAb | CST | 2764S |
| CHOP | Mouse mAb | NovusBio | 1335SS |
| XBP-1S | Mouse mAb | BioLegend | 658802 |
| β-actin | Mouse mAb | CST | 3700S |

| **Antibody**  (for HHC) | **Source** | **Company** | **Cat. #** | **Retrieval** | **Dilution** |
| --- | --- | --- | --- | --- | --- |
| BiP | Rabbit mAb | Abcam | 66138 | Citrate buffer | 1:300 |
| Calnexin | Rabbit mAb | CST | 2976 | Citrate buffer | 1:50 |
